# Reply: Beyond the ECG: Toward Patient-Centered Digital Triage for Acute Coronary Syndromes

**DOI:** 10.1016/j.jacadv.2026.103138

**Published:** 2026-08-12

**Authors:** Alexei Shvilkin

**Affiliations:** Cardiovascular Division, Department of Medicine, Beth Israel Deaconess Medical Center, Boston, Massachusetts, USA

We thank Dr Skalidis and colleagues for their thoughtful comments and for emphasizing the central problem that motivated our study: persistent patient-related delays in evaluating possible acute coronary syndrome (ACS).^1^ We agree that digital tools combining patient-specific clinical information with self-acquired electrocardiogram (ECG) data may help patients recognize risk earlier and seek appropriate care.

A key premise of our approach is that the ECG, regardless of the number of leads, recording platform, or analytic sophistication, has intrinsic limitations for ACS diagnosis, particularly outside clear ST-segment elevation. In an era of enthusiasm for artificial intelligence applied to ECG interpretation, it is tempting to assume that better prehospital ACS triage will come from extracting more information from the signal itself. We believe this view is too narrow. The relevant question is not how many leads, features, or neural-network layers are required to diagnose ACS from the ECG, but how ECG findings should be interpreted in the context of the patient in whom they were recorded.

This reflects traditional bedside diagnosis. A physician listens to the patient’s symptoms, analyzes the details of the complaint beyond the blanket label of “chest pain,” considers demographic and historical risk, and only then interprets the ECG and other tests. Diamond and Forrester^2^ formalized the concept that diagnostic tests gain meaning only after estimating pretest probability from clinical variables. Although ACS triage differs from stable coronary disease assessment, the principle remains relevant.

Efforts to improve prehospital ACS evaluation should therefore expand beyond better ECG acquisition or automated interpretation alone. Structured clinical data should be treated not as an accessory to the ECG, but as a central component of risk assessment, especially in non-ST-segment elevation ACS. Properly designed digital tools may help reproduce the disciplined reasoning of an initial prehospital or outpatient physician evaluation and advance it toward the patient’s own home environment.

Skalidis et al correctly note that our study enrolled patients after presentation to an emergency department, with structured clinical and ECG data acquisition. This design allowed standardized data collection and adjudicated outcomes, but it does not fully reproduce independent patient use. Future studies should evaluate symptom entry, ECG quality, interpretability, failure modes, and model calibration in older patients, anxious patients, and those with limited digital literacy.

We agree that a prospectively stored baseline ECG would be preferable to the postevent reference ECG used in our analysis. However, obtaining true pre-event recordings presents logistic challenges when a device has not yet been approved and deployed for this indication. The postevent reference ECG therefore served as a pragmatic within-patient comparator.

Finally, false-positive and false-negative trade-offs must be evaluated prospectively and separately across ACS phenotypes. This study should be viewed as an early step toward patient-centered digital ACS risk assessment, with real-world validation required before clinical implementation.
